# Progress in Water, Sanitation and Hygiene (WASH) coverage and potential contribution to the decline in diarrhea and stunting in Ethiopia

**DOI:** 10.1111/mcn.13280

**Published:** 2021-11-04

**Authors:** Meron Girma, Alemayehu Hussein, Tom Norris, Tirsit Genye, Masresha Tessema, Anne Bossuyt, Mamuye Hadis, Cornelia van Zyl, Kitka Goyol, Aregash Samuel

**Affiliations:** ^1^ National Information Platforms for Nutrition (NIPN) Ethiopian Public Health Institute Arbegnoch Street Addis Ababa 1242 Ethiopia; ^2^ National Information Platforms for Nutrition (NIPN) Collaborator International Food Policy Research Institute Addis Ababa Ethiopia; ^3^ National Information Platforms for Nutrition (NIPN) International Food Policy Research Institute Addis Ababa Ethiopia; ^4^ Knowledge Translation Directorate Ethiopian Public Health Institute Addis Ababa Ethiopia; ^5^ Water, Sanitation and Hygiene (WASH) UNICEF Addis Ababa Ethiopia

**Keywords:** children, diarrhoea, Ethiopia, hygiene, sanitation, stunting, water

## Abstract

Inadequate safe water supply and poor sanitation and hygiene continue to be important risk factors for diarrhoea and stunting globally. We used data from the four rounds of the Ethiopian Demographic and Health Survey and applied the new World Health Organization (WHO)/UNICEF Joint Monitoring Program (JMP) service standards to assess progress in water, sanitation and hygiene (WASH) coverage between 2000 and 2016. We also performed an age‐disaggregated pooled linear probability regression analysis followed by a decomposition analysis to determine whether changes in WASH practices have contributed to the changing prevalence of diarrhoea and stunting in children under 5 years of age. We observed a significant increase in the coverage of safe drinking water and adequate sanitation facilities over the period. At the national level, the use of a basic water source increased from 18% in 2000 to 50% in 2016. Open defecation declined from 82% to 32% over the same period. However, in 2016, only 6% of households had access to a basic sanitation facility, and 40% of households had no handwashing facilities. The reduction in surface water use between 2000 and 2016 explained 6% of the decline in diarrhoea observed among children aged 0–5 months. In children aged 6–59 months, between 7% and 9% of the reduction in stunting were attributable to the reduction in open defecation over this period. Despite progress, improvements are still needed to increase basic WASH coverage in Ethiopia. Our findings showed that improvements in water and sanitation only modestly explained reductions in diarrhoea and stunting.

**Key messages**

- The coverage of safe and adequate drinking water and sanitation facilities significantly increased between 2000 and 2016.
- Less than 10% of households have access to basic hygiene facilities. This highlights the need for interventions that increase access to basic hygiene.
- Improvements are still needed to increase coverage of higher WASH standards and to address prowealthy and prourban inequalities in WASH coverage.
- Improvements in water and sanitation services contributed to only modest reductions in the probability of experiencing diarrhoea and stunting.

## 1 INTRODUCTION

Despite substantial progress in reducing the burden of diarrhoeal diseases globally, diarrhoea remains one of the leading causes of mortality in children under 5 years of age, and the second leading infectious cause of mortality in this age group, behind lower respiratory infections (Troeger et al., 2017, 2018, 2020). For example, in 2016, it was estimated that diarrhoea was responsible for approximately 9% of all deaths in children aged under 5 years (Troeger et al., 2018). In addition, diarrhoeal disease is also a major cause of disability‐adjusted life years (DALYs), and in particular, it is a major contributor to the high rates of stunted growth observed in low‐income settings (Danaei et al., 2016).

Inadequate safe water supply and poor sanitation and hygiene are significant risk factors for diarrhoea‐related morbimortality globally (Forouzanfar et al., 2015). For example, it has been estimated that 94% of under‐5 deaths in 2017 could have been averted by the provision of safe water, sanitation and hygiene (WASH) (Troeger et al., 2020). Inadequate water (e.g., water supplied from unprotected springs and dug wells, surface water and water tanks) and sanitation (flush toilets not connected to a sewer/septic system, pit latrines without slab, bucket latrines or open defecation) practices increase faecal–oral transmission of pathogens, which subsequently leads to diarrhoea (defined as more than three liquid stools per day) (Lutter et al., 2018). Repeated episodes of diarrhoea lead to loss of nutrients and fluids, causing overall weakness and dehydration. In addition, ingestion of faecal bacteria in large quantities leads to environmental enteric dysfunction (EED), a subclinical condition of the small intestine characterized by blunted villi, intestinal inflammation, increased intestinal permeability and consequently a reduced nutrient absorption (Humphrey, 2009) and increased likelihood of undernutrition and stunted physical and cognitive development.

Unsurprisingly therefore, it has been shown that diarrhoeal diseases disproportionately affect settings with poor access to health care, safe water and sanitation, and low‐income or marginalized populations (Troeger et al., 2017). In Ethiopia, for example, where recent estimates reveal that <15% of the population have access to safely managed water supplies and <10% using safely managed sanitation facilities and basic hygiene facilities, diarrhoeal disease remains a major public health concern (World Health Organization [WHO]/UNICEF Joint Monitoring Program [JMP], 2021). In 2016, estimates revealed a prevalence of 12%, and 13% of child deaths were attributable to diarrhoea (Central Statistical Agency [Ethiopia] and ICF, 2016). This high burden of diarrhoea is also likely contributing to the high (but decreasing) stunting burden in Ethiopia, with recent estimates revealing a stunting prevalence of 37% (Ethiopian Public Health Institute [EPHI] [Ethiopia] and ICF, 2019).

The positive effect of improved WASH practices and a reduced diarrhoea incidence in young children is well known and has been reported in several meta‐analyses (Clasen et al., 2007; Fewtrell et al., 2005; Wolf et al., 2014). These findings have led to the hypothesis that, via the positive effect on diarrhoea risk, WASH may also confer benefits to linear growth. Although observational and quasi‐experimental studies have shown support for an association between WASH practices and child height (Garcia et al., 2013; Lin et al., 2013; Masibo & Makoka, 2012; Merchant et al., 2003; Spears et al., 2013) (Dangour et al., 2013), two large WASH Benefits trials (Luby et al., 2018; Null et al., 2018) and the SHINE trial (Humphrey et al., 2019) have cast doubt on this association, with all three trials observing no effect on linear growth (LAZ) at 18–24 months of age, with the authors concluding that elementary household‐level WASH interventions are unlikely to improve child growth (Pickering et al., 2019).

Although the evidence regarding the efficacy of WASH interventions on reducing childhood stunting is equivocal, the benefits of promoting improved WASH practices remain important for child nutrition (e.g., wasting and micronutrient deficiencies), health and development. The Sustainable Development Goals (SDGs) recognize WASH as central to sustainable development with SDG 6 calling for universal access to safe and adequate water, sanitation and hygiene for all by 2030 (United Nations, 2015). There is, however, a lack of evidence regarding the trends and predictors of WASH practices over time in Ethiopia and thus little indication about the progress the country is making in achieving SDG 6. The study by Beyene and colleagues, conducted in 2014, assessed the trends in sanitation practices only and revealed that more than half of the population still used unimproved sanitation facilities. A more recent cross‐sectional study based on data from 2016 assessed access to and factors associated with improved drinking water sources and toilet facilities (Andualem et al., 2021). The proportion of households' access to improved drinking water sources and toilet facilities was 70% and 25%, respectively, with several factors, including wealth index, distance to water source and sex of household head, related to access. However, the cross‐sectional study design meant it was not possible to assess progress in WASH trends over time.

The aim of this study therefore was to describe trends in WASH service coverage between 2000 and 2016 and investigate whether any changes in WASH practices have contributed to a decline in the prevalence of diarrhoea and stunting in children under 5 years of age in Ethiopia.

## 2 METHODS

### 2.1 Data source

We used data from the four rounds (2000, 2005, 2011 and 2016) of the EDHS (Central Statistical Agency [Ethiopia] and ICF, 2011, 2016; Central Statistical Agency [Ethiopia] and ORC Macro, 2001, 2006) for this analysis. These surveys are standardized across rounds and collect nationally and regionally representative cross‐sectional data for households, children under 5 years of age, women of reproductive age and men. For this analysis, we used the household and child datasets of the EDHS.

### 2.2 Trends in WASH practices: WASH indicators

We used the new WHO/UNICEF JMP WASH service standards (WHO/UNICEF Joint Monitoring Program [JMP], 2019) to describe WASH service coverage in Ethiopia using EDHS data. These new WASH service ladders (described in Table S1) build on the widely used improved/unimproved facility type classification and introduce additional indicators to reflect higher standards (WHO/UNICEF Joint Monitoring Program [JMP], 2019). Data were not available to construct ‘safely managed water’ and ‘sanitation standards’. Consequently, the highest service standards used in this analysis are ‘basic drinking water facilities’ and ‘basic sanitation facilities’. A basic drinking water source refers to drinking water from an improved source, with collection time, not more than 30 min for a round trip. A basic sanitation facility is an improved toilet facility that is not shared with other households. The highest hygiene service standard is basic, which requires the availability of a handwashing facility with soap and water on the premises.

### 2.3 Exposures

As data pertaining to hygiene practices were only available in 2011 and 2016, the analysis to assess the association between WASH practices with outcomes focused on drinking water and sanitation practices only. Specifically, the exposures of interest were the use of surface water as a drinking source and the practice of open defecation. Information on the type of water facility used was reported, whereas the type of sanitation facility used was assessed through a combination of report and observation.

### 2.4 Outcomes

The primary outcome of our regression analysis was diarrhoea prevalence, the results of which are reported in the main text. Stunting prevalence was our secondary outcome, and this analysis is presented as supplementary analyses. Diarrhoea was defined as the percentage of children with diarrhoea (three or more loose stools per day) at any time in the 2 weeks preceding the survey. Stunting was defined as HAZ/LAZ below −2 SD of the median based on the WHO 2006 Child Growth Standards (de Onis et al., 2004).

### 2.5 Covariates

The relationships between exposures and outcomes were adjusted for covariates, which were selected on the basis of the Lancet framework for action (Black et al., 2013) or previously reported associations with the outcome variables (Argaw et al., 2019; Danaei et al., 2016; Dearden et al., 2017; Headey & Hoddinott, 2015; Headey & Palloni, 2019; Headey et al., 2016, 2017; Oswald et al., 2017; Troeger et al., 2018). These included maternal and paternal education, household wealth, maternal employment, maternal height, child age, child sex, residence, region and survey round.

### 2.6 Statistical analysis

To account for the cluster sampling used in the EDHS, sampling weights were applied to estimate WASH service coverage, and the prevalence of stunting and diarrhoea across survey rounds. We used stacked area plots, produced at the national and regional level, to explore changes in WASH practices between 2000 and 2016. Additionally, equity plots documenting the temporal changes in WASH practices by wealth quintile and place of residence (urban vs. rural) were produced. We computed the slope index of inequality (SII) to quantify the absolute difference (percentage point) in WASH service coverage between the wealthiest and the poorest households. Greater SII values represent higher levels of inequality. The SII is equal to zero if there is no inequality. To account for the incidence of diarrhoea and the timing of growth faltering in childhood, we made an a priori decision to investigate the relationship between WASH practices and diarrhoea and stunting using an age‐disaggregated approach. Accordingly, stratified analyses were performed in the following age ranges: 0–5, 6–11, 12–23 and 24–59 months. In addition, we made an a priori decision to include in the analysis, for households with more than one child below 59 months, only the youngest child with anthropometric data. Within the age groups listed above, we performed a regression decomposition analysis, which seeks to determine the contribution made by changes in the mean levels of exposures over time to changes in outcomes. Specifically, the decomposition analysis sought to reveal whether changes in surface water usage and open defecation practices identified in the previous step contributed to observed reductions in diarrhoea and stunting between 2000 and 2016. As has been done in other decomposition analyses, we initially examined the relationship between diarrhoea and stunting with the exposures by pooling data from all rounds of the EDHS and performing a linear probability regression analysis (adjusted for the covariates listed above and with robust standard errors to account for clustering) (Headey et al., 2015, 2016, 2017). A key assumption underpinning the use of a decomposition analysis, which is based on a pooled regression model, is that coefficients are time invariant (i.e., the magnitude and direction of coefficients are stable over time). In order to test this assumption, we performed a series of Chow tests, which test whether coefficients differ significantly over time. We did not find support for the coefficients being time‐varying, and we therefore followed the approach by Headey and Hoddinott (2015) and Headey et al. (2016, 2017) and performed a simple decomposition analysis for exposures that were associated with outcomes in the regression analysis at 10% level of significance. The decomposition equation takes the form of

$$∆{\bar{N}}_{i,t}=\beta \left({\bar{X}}_{t=k}-{\bar{X}}_{t=1}\right),$$

where *t* = 1 represents the 2000 EDHS, *t* = *k* is the 2016 EDHS, and *β* and $\bar{X}$ represent the regression coefficient and sample mean, respectively, for a given variable. The decomposition then entails multiplying observed changes in the means of each variable over time by its regression coefficient. Doing so gives the predicted change in the probability of diarrhoea or stunting from each change in a selected variable and thus shows the estimated contributions of each variable to changes in diarrhoea and stunting. For example, let us assume that the practice of open defecation decreased by 20% between 2000 and 2016 and that the regression coefficient, in a model with diarrhoea as the outcome, comparing the practice of open defecation with no open defecation is 0.05. If we multiply these two numbers, we will get 1%, indicating that the changes in the practice of open defecation account for 1% decline in diarrhoea. If diarrhoea declined by 5% over the same period, changes in the prevalence of basic toilet facilities would therefore represent a 20% contribution to the reduction in diarrhoea prevalence. Data management and statistical analysis were conducted in stata Version 14.0.

## 3 RESULTS

Data from 61,715 households across the four rounds of the EDHS (2000–2016) were used to describe trends in WASH practices. After exclusion of children with missing values for outcomes, exposures and covariates, 20,509 children were included in the diarrhoea model and 20,408 were included in the stunting model for the pooled regression and decomposition analysis. The mean (SD) child age was 22 (15) months, and 51% of the children were male. Characteristics of the study population are shown in Table 1.

**Table 1:** Characteristics of study population included in the analysis

Variables	2000 (*n* = 6373)	2016 (*n* = 6323)	Mean/percentage change (2016–2000)	*p* value for mean/percentage change
Children (0–59 months)				
Child age (months), mean (SD)^a^	22 (15.1)	22 (15.6)	0	0.405
Child: female (%)	49	49	0	0.949
Prevalence of diarrhoea (%)	29	15	−14	<0.001
Prevalence of stunting (%)	55	33	−22	<0.001
Maternal and paternal characteristics				
Maternal height (cm), mean (SD)	156 (7.1)	157 (6.6)	1	<0.001
Maternal formal education^a^ (%)	17	36	19	<0.001
Paternal formal education^a^ (%)	35	53	18	<0.001
Maternal employed^b^ (%)	65	46	−19	<0.001
Household characteristics				
Household surface water use (%)	37	12	−25	<0.001
Household open defecation (%)	86	35	−51	<0.001
Wealth				
Poorest (%)	21	21	0	0.993
Poorer (%)	21	22	1	0.362
Middle (%)	21	21	0	0.694
Richer (%)	20	19	−1	0.18
Richest (%)	16	16	0	0.97

^a^ Attended any level of formal education.

^b^ Women who worked in the 12 months preceding the survey or are currently working.

### 3.1 Progress in WASH coverage

**Figure 1:**
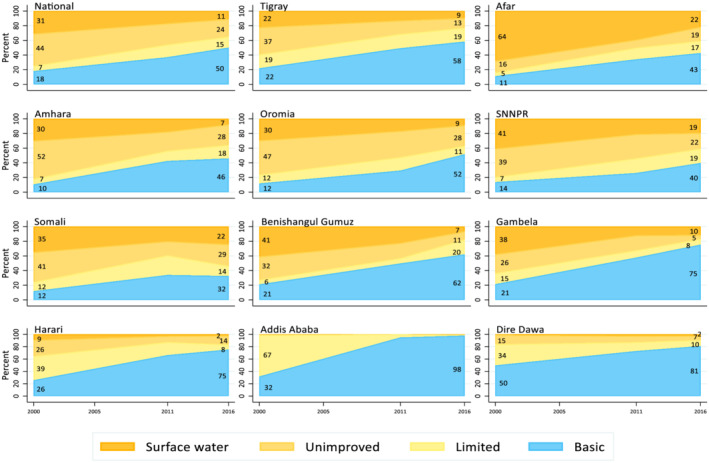
Changes in water coverage in Ethiopia between 2000 and 2016: National and regional levels. Stacked area charts show change in water service standards coverage nationally and subnationally (11 regions) between 2000 and 2016. The water service standards used were ‘Basic’: drinking water from an improved source, provided collection time is not more than 30 min for a round trip, including queuing; ‘Limited’: drinking water from an improved source, which collection time exceeds 30 min for a round trip, including queuing; ‘Unimproved’: drinking water from an unprotected dug well or unprotected spring; and ‘Surface water’: drinking water directly from a river, dam lake, pond, stream, canal or irrigation canal

Figure 1 shows the progress in water coverage between 2000 and 2016 nationally and regionally. In 2016, half (50%) of households in Ethiopia used a basic water source. This figure represents an increase from 2000, when only 18% of households used a basic water source. Despite this progress, in 2016, 15% of households still used a limited water source (an improved water source with a collection time of more than 30‐min round trip), 24% used an unimproved water source and 11% used surface water. Although improvements in water service standards were seen across all regions, progress was uneven with some regions achieving higher coverage compared with others.

**Figure 2:**
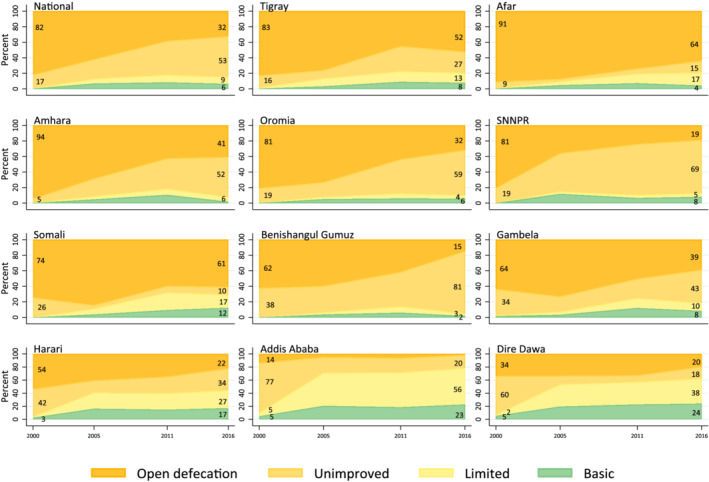
Changes in sanitation coverage in Ethiopia between 2000 and 2016: National and regional levels. Stacked area charts show change in sanitation service standards coverage nationally and subnationally (11 regions) between 2000 and 2016. The sanitation service standards used were ‘Basic’: use of improved facilities that are not shared with other households; ‘Limited’: use of improved facilities shared between two or more households; ‘Unimproved’: use of pit latrines without slab or platform, hanging latrines or bucket latrines; and ‘Open defecation’: disposal of human faeces in fields, forests, bushes, open bodies of water, other open spaces or with solid waste

**Figure 3:**
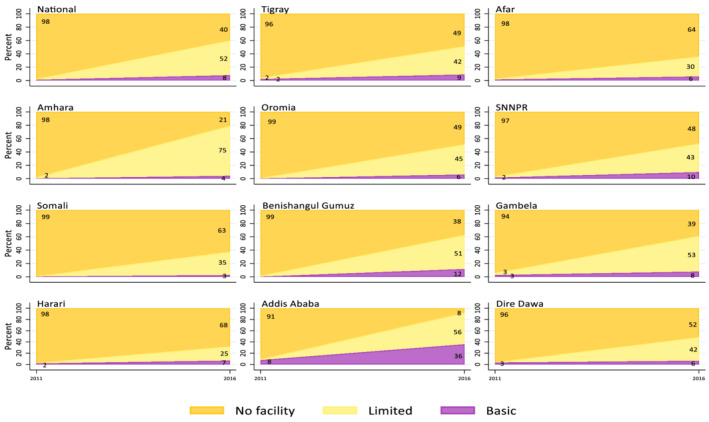
Changes in hygiene coverage in Ethiopia between 2011 and 2016: National and regional levels. Stacked area charts show change in hygiene service standards coverage nationally and subnationally (11 regions) between 2011 and 2016. The hygiene service standards used were ‘Basic’: availability of a handwashing facility on premise with soap and water; ‘Limited’: availability of a handwashing facility on premise without soap and water; and ‘No facility’

Figure 2 shows trends in sanitation service coverage. Although some progress has been made since 2000, large gaps remain in coverage of sanitation facilities. In 2016, only 6% of Ethiopian households used a basic sanitation facility. Open defecation showed a significant decline between 2000 and 2016. In 2000, 82% of households practised open defecation compared with 32% in 2016. Similar to water coverage, regional differences in the type of sanitation facilities used were observed. The largest percentages of households that used basic sanitation facilities were in Addis Ababa and Dire Dawa, both city administrations. The percentage of households that still practised open defecation was still high in some regions (Afar [64%], Somali [61%] and Tigray [52%]). The use of basic handwashing facilities is low in Ethiopia. In 2016, only 8% of households had a handwashing facility with soap and water available on the premises (Figure 3). Additionally, 40% had no handwashing facility on the premises.

### 3.2 Trends in inequalities in basic WASH standard coverage

**Figure 4:**
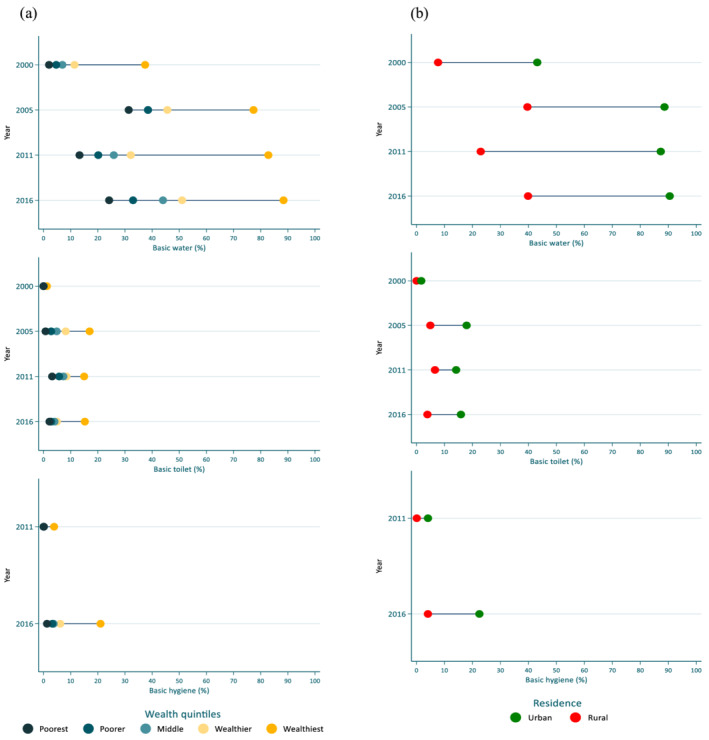
Change over time in basic water, basic sanitation and basic hygiene coverage by wealth and residence. Progress in basic water and basic sanitation coverage disaggregated by (a) wealth quintiles between 2000 and 2016 and (b) residence between 2000 and 2016

In general, the use of a basic water facility increased over time across all wealth quintiles. However, the change was much greater in the wealthiest quintile, increasing from less than 40% of households in 2000 to just under 90% in 2016. As such, the gap between the poorest and the richest households (represented by the width of the bars shown in Figure 4 and quantified using the SII) increased between 2000 and 2016. The absolute differences in the basic water coverage between the poorest and richest households (SII) were 32%, 33%, 59% and 63% in 2000, 2005, 2011 and 2016, respectively. When disaggregated by place of residence, the proportion of households using a basic water facility was consistently greater in urban areas, and this difference increased between 2000 and 2016. In both urban and rural settings, the proportion of households using a basic water facility increased markedly between 2000 and 2005, with little change observed thereafter. Access to basic sanitary facilities was consistently higher among the wealthiest households compared with the poorest households, though these differences were small compared with differences seen for access to basic water facilities. As expected, wealthier households and households in urban areas had more access to basic hygiene facilities compared with poorer households and households located in rural areas, respectively, and this difference increased between 2011 and 2016.

### 3.3 Trends in diarrhoea and the relationship with WASH practices

The prevalence of diarrhoea declined during this period. Reductions in the prevalence of diarrhoea between 2000 and 2016 were observed, declining from 29% to 15% in children under 5 years of age, with the largest reduction observed in children aged 12–23 months (38% in 2000 to 19% in 2016; a 19% change). Across all EDHS rounds, the prevalence of diarrhoea was lowest in the youngest infants (0–5 months). We used pooled regression analysis results (2000–2016) (see Table S4) and the per cent change in surface water use and open defecation to estimate the contribution of the reduction in these practices to the decline in diarrhoea. Among children aged 0–5 months, the use of surface water was associated with a 4% (95% confidence interval [CI]: 1%, 7%, *p* = 0.018) increase in the probability of diarrhoea (Table S4). Similarly, children who lived in households that practice open defecation had a 3% (95% CI: 0, 6%, *p* = 0.087) higher probability of diarrhoea. In contrast, neither household surface water use nor open defecation was associated with the probability of stunting. In children aged 24–59 months, the practice of open defecation was associated with a 3% (95% CI: 1%, 5%, *p* = 0.012) increase in the probability of diarrhoea and stunting (*β*: 3%, 95% CI:0%, 6%, *p* = 0.064). The reduction in surface water use between 2000 and 2016 explained 6% of the decline in probability of experiencing diarrhoea (at any time in the 2 weeks preceding the survey) among children aged 0–5 months and 7% of the decline among children aged 24–59 months (Figure 4). Additionally, the reduction in open defecation explained between 7% and 8% of the decrease in diarrhoea among children aged 0–5 and 24–59 months.

#### 3.3.1 Supplementary analysis

**Figure 5:**
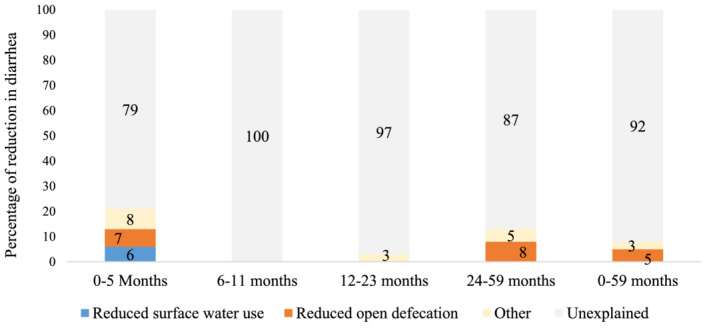
Estimated contribution of the decline in surface water use and the practice of open defecation between 2000 and 2016 to the decline in diarrhoea observed over the same period. Per cent contribution of the change in surface water use and open defecation to the decline in diarrhoea between 2000 and 2016. The per cent contribution of factors was estimated using simple decomposition in which changes in the percentages of the exposures and covariates are multiplied by the pooled regression coefficients. Other category includes maternal and paternal education, household wealth, maternal employment, child age, child sex, residence, region and survey round

In all children aged 0–59 months, stunting declined by 22% between 2000 (55%) and 2016 (33%). The prevalence of stunting was lowest in infants aged 0–5 months and increased with age, with the highest prevalence in children aged 24–59 months (Figure 5).

Open defecation explained some of the reduction in stunting in all age groups except 0–6 months. The substantial reduction in open defecation between 2000 and 2016 explained between 7% and 9% of the decline in stunting in children aged 6–11, 12–23 and 24–59 months. In contrast, the reduction in surface water use was not significantly associated with the probability of stunting across all age groups.

## 4 DISCUSSION

This study utilized data from multiple rounds of the EDHS to derive trends in WASH practices between 2000 and 2016 and relate these trends to changes in the prevalence of diarrhoea and stunting among Ethiopian children aged 0–59 months. We observed a significant increase in the coverage of safe and adequate drinking water and sanitation facilities over the period. Despite these positive results, the finding that only 6% of households had access to basic sanitation facilities and 40% of households had no handwashing facilities at all means that more efforts are still required to improve WASH coverage in Ethiopia. The improvements in WASH practices contributed to some of the observed reductions in the probability of experiencing diarrhoea and stunting. The reduction of surface water use over this period explained 6% of the decline in diarrhoea observed among children aged 0–5 months.

We present original findings regarding the change in age‐disaggregated diarrhoea prevalence in children under 5 years of age. A recent systematic review of 31 studies published between 2003 and 2017 estimated the prevalence of diarrhoea in children under 5 years at 22% (Alebel et al., 2018). Our analysis, by estimating the prevalence in age‐disaggregated groups, provides greater insight into the burden of and risk factors associated with diarrhoea in early childhood. We observed the lowest prevalence of diarrhoea in those aged 0–5 months. Thereafter, we reveal a pattern of an increasing burden of diarrhoea around the time of the introduction of complementary feeding at 6 months and a subsequent reduction after 12 months. Taken together, these findings highlight the benefits of exclusive breastfeeding and the increased risk of diarrhoea associated with the transition to complementary feeding, likely a consequence of the consumption of contaminated food and water as a result of poor WASH conditions. This finding of an increasing burden of diarrhoea at this age has been reported in Ethiopia (Central Statistical Agency [Ethiopia] and ICF, 2016; Central Statistical Agency [Ethiopia] and ORC Macro, 2001; Golan et al., 2019) and in other low‐ and middle‐income (LMIC) countries settings (Victora & Barros, 2000). In light of the above, we observed an unexpected association with the probability of diarrhoea and the type of water source (surface water) among children aged 0–5 months. This finding is surprising because we would expect the use of surface water to increase the risk of diarrhoea once complementary feeding starts after 6 months. The lack of association between diarrhoea and surface water use in older children seen in this analysis supports findings from recent WASH trials conducted in Bangladesh (Luby et al., 2018), Zimbabwe (Humphrey et al., 2019) and Kenya (Null et al., 2018), which observed little effect of water treatment interventions on diarrhoea incidence in children. In terms of sanitation practices, we observed that 7% and 8% of the estimated decline in diarrhoea among children aged 0–5 and 24–59 months, respectively, were attributed to the reduction in open defecation. This observed stronger association between sanitation and diarrhoeal incidence compared with water usage has also been reported in an analysis of data from 217 Demographic and Health Surveys (Fuller et al., 2015) as well as a difference‐in‐difference analysis done by Headey and Palloni (2019).

Ethiopia has made substantial progress in its coverage of safe and adequate drinking water and sanitation facilities. The first campaign for universal sanitation was implemented in the SNNPR region in 2003 (Bibby & Knapp, 2007). The campaign was reliant on a strong commitment and participation from all levels of the community, including a network of volunteer health promoters at the village level, environmental health workers at the woreda level and strong support from the regional health bureau (Bibby & Knapp, 2007). Rapid improvement was seen in just 2 years, with pit latrine ownership rising from below 13% in 2003 to 88% in 2006 (Bibby & Knapp, 2007). Following this success, further campaigns based upon community engagement and behavioural change were developed, culminating in the implementation of Community‐Led Total Sanitation and Hygiene (CLTSH) approach. This was formally adopted by the Federal Ministry of Health (FMoH) in 2011 with subsequent roll out via the Health Extension Program (HEP), a community‐based strategy aimed at delivering health promotion, disease prevention and selected health services at the community level (Federal Democratic Republic of Ethiopia: Ministry of Health, 2012). The widespread rollout of the CLTSH is a key factor in the country demonstrating one of the fastest reductions in open defecation globally (Novotný et al., 2018).

Despite the progress seen, improvements are still needed as the quality of WASH services is low (only 6% have basic sanitation facilities and 40% do not have handwashing facilities). Furthermore, inequalities in WASH coverage still persist, with wealthier households and those residing in urban settings more likely to have access to better water and sanitation facilities. We also reveal persisting regional disparities in WASH coverage and in the Afar, Somali and Tigray regions, where open defecation is still predominant. It has been reported that there is comparatively low commitment to planning for open defection free outcomes in these regions and that training materials and manuals for CLTSH are not translated into local languages (UNICEF, 2017). Although the reduction in the practice of open defecation has been marked, this has typically been achieved by the construction of low quality ‘unimproved’ sanitation facilities, with only small increases in ‘limited’ and ‘basic’ sanitation facilities in most regions. This lack of assistance and sanitation technology required for the progression up the sanitation ladder has been suggested as one potential impeding factor preventing the effective implementation of CLTSH (Tessema, 2017). Other factors include lack of effective social mobilization techniques (e.g., via a command and control approach rather than ‘facilitated ignition’) (Novotný et al., 2018), inadequate participation of females (at the household level, females have been shown to drive access to improved sanitation and water facilities) (Bibby & Knapp, 2007) (Andualem et al., 2021) and support of poor families, and a lack of training for in‐depth understanding of the participatory appraisal tools (Tessema, 2017). A national evaluation of the implementation and impacts of the CLTSH programme concluded that implementation has often been inadequate in terms of quality and that the programme has not improved latrine sanitation levels enough to sustain positive sanitation and hygiene behaviours (UNICEF, 2016). This is supported by evidence suggesting that within 1 and 2 years of achieving open defecation free (ODF) status, many ODF villages lose this certification (Federal Democratic Republic of Ethiopia: Ministry of Health, 2012). To address the lack of assistance and sanitation technology as an impediment to further improving sanitation practices, the Sanitation Marketing Initiative was launched in 2013 by the FMoH with the aim of increasing access to affordable and improved basic sanitation and hygiene products (Federal Democratic Republic of Ethiopia: Ministry of Health, 2013). Since its initiation, regional governments have implemented sanitation marketing and business development interventions in selected district health offices. The limited evidence available regarding the efficacy of the initiative suggests that it may be associated with improved latrine quality and presence of handwashing facilities (Vrana et al., 2017). Another initiative that has been established in Ethiopia with the aim of addressing the challenges faced by the WASH sector is the ONEWASH National Program (Federal Democratic Republic of Ethiopia, 2013). The overarching objective of the programme is to achieve universal, sustainable, climate resilient and equitable access to safe and affordable water for all, along with improved, low environmental impact, sanitation. To achieve this, the programme has multiple medium and longer term goals, which include the construction and rehabilitation of water supply schemes and sanitation facilities in rural and urban areas, health institutions and schools, improving the resilience of WASH services to climatic shocks through climate‐adaptive service delivery and improving water resource monitoring and planning to assess climate change risks (Federal Democratic Republic of Ethiopia, 2013).

Despite significant improvements in water coverage alongside a significant reduction in open defecation, our study revealed that these were only associated with modest reductions in diarrhoea and stunting in Ethiopian children age 0–59 months. Results from other decomposition analyses have also reported only modest benefits (D. Headey et al., 2016, 2017). Furthermore, other studies such as the Malnutrition and the Consequences for Child Health and Development (MAL‐ED) birth cohort (Kosek & Mal‐Ed Network Investigators, 2017) and WASH Benefit and SHINE trials (Pickering et al., 2019) have found little to no effects of improved WASH and EED and on stunting and diarrhoea. A possible reason for the lack of relationship is that neighbourhood WASH practices are more important than household practices for the reduction of environmental faecal contamination (Jung et al., 2017). However, in a supplementary analysis replacing household WASH practices with community practices, this lack of association persisted (data not shown). In light of the modest results of improvements in WASH on diarrhoea and stunting, there have been calls for research to identify interventions, labelled ‘Transformative WASH’, that radically reduce faecal contamination in the household environment in LMIC (Pickering et al., 2019). Proposed interventions may include high community coverage of improved sanitation facilities (Fuller & Eisenberg, 2016), complete separation of animal faeces from people's living environments (Boehm et al., 2016; Mbuya et al., 2015), continuous and convenient access to uncontaminated water (Pickering & Davis, 2012) and reductions in faecal contamination on surfaces where young children crawl and play (Ercumen et al., 2018). Given that in 2016, 40% of households had no hygiene facilities, 53% rely on unimproved sanitation facilities, and about 35% of households rely on unimproved water sources or surface water, much more work is needed to increase coverage of even basic WASH services across Ethiopia.

Our finding of an overall reduction in the stunting prevalence in children under 5 years of age in Ethiopia has been reported elsewhere (Golan et al., 2019; Takele et al., 2019). However, the age‐disaggregated analysis has enabled us to also identify a changing dynamic of age‐related stunting prevalence over time. For example, in 2000, the prevalence of stunting increased immediately after birth (0–5 months) and continued to increase thereafter. In 2016, however, in addition to numbers being lower overall, the time at which the stunting prevalence increased rapidly appears to be later than in 2000, with little change in stunting prevalence in infants aged 0–5 months (13%) and 6–11 months (17%), followed by a rapid rise between 12 and 23 months (39%). This delay in growth faltering may be attributable to improved breastfeeding practices in the first 6 months and improved complementary feeding practices at 6 to 12 months, which may have reduced the exposure to infections as a result of poor WASH conditions.

Although we did not find an association between surface water use and stunting, open defecation was associated with an increased probability of stunting across the age groups. A lower risk of stunting with improved sanitation but not improved water has been reported for Ethiopian children elsewhere (Dearden et al., 2017). The mixed findings between WASH practices and stunting observed in our study are mirrored in the mixed evidence reported in the literature, with observational studies reporting positive associations between WASH practices and linear growth in childhood (Garcia et al., 2013; Lin et al., 2013; Masibo & Makoka, 2012; Merchant et al., 2003), whereas results from recent RCTs suggest no effect of WASH interventions on LAZ‐scores at 18–24 months of age (Humphrey et al., 2019; Luby et al., 2018; Null et al., 2018). Our findings showed that the observed reduction in open defecation contributed to 9% and 7% of the decline in stunting seen among children aged 6–11 and 12–59 months, respectively. Because one proposed pathway between poor WASH practices and stunting is via increase in the incidence of diarrhoea (and EED), our findings of increased probability of diarrhoea and stunting with open defecation are expected.

We have utilized nationally representative data to identify the contributions made by WASH practices to reductions observed in diarrhoea and stunting. We constructed age‐disaggregated regression models that have several benefits. First, such an approach has the benefit of being able to identify differential contributions made by variables in specific age periods and thus periods in which intervening on a particular variable may be more advantageous. For example, infants under 2 years represent an age group of particular interest, as most growth faltering takes places in the first 1000 days of life. Second, because age‐disaggregated models respect the changing age dynamics of growth faltering observed across childhood (Alderman & Headey, 2018), they are less likely to underestimate the effects of any factor on stunting. In terms of limitations, due to the cross‐sectional nature of the data, causal inferences cannot be made about the relationship between exposure and outcome variables. A second limitation is that we were unable to assess the contribution of the use of higher quality WASH services to the decline in diarrhoea and stunting. The EDHS relies predominantly on self‐reports or proxy reports, which are therefore subject to recall bias and which may affect older children to a greater extent than younger ones. Reliance on the recall may have also led to the underestimation of diarrhoea prevalence if, for example, respondents omit to mention episodes of diarrhoea that did not result in the utilization of medical care or medicines. Lastly, the lack of data on additional predictors of diarrhoea and stunting meant that our models only explained a small portion of the change in outcome variables.

Given the persistence of inequalities in WASH coverage, particularly regional disparities, we advocate a reconsideration of the CLTSH to improve suitability across all regions, whether this would be greater investment in the HEP in these regions or at a more basic level, ensuring that CLTSH materials are in an accessible format. We do acknowledge however that due to the nomadic/semipastoralist nature of some of the communities in these regions, actioning change in these settings may be a challenge. Given the greater time spent by females performing WASH‐related activities, it is perhaps unsurprising that female sex and female empowerment have been associated with improved household WASH practices in Ethiopia (Bogale, 2021) and elsewhere (Dickin et al., 2021). The continued empowerment and involvement of females in WASH initiatives should therefore be promoted. Finally, continued promotion of the Sanitation Marketing Initiative and engagement of the private sector will increase financial investment in the WASH sector and improve access to affordable and improved basic sanitation and hygiene products. Given that cost–benefit analyses show that for each $1 invested in WASH results in a return of between $2 and $9 via the benefits conferred by WASH interventions (WHO, 2012) on health, social and environmental well‐being, investment is likely to result in substantial long‐term economic benefit to the country.

In conclusion, the current analysis has highlighted the progress made in WASH practices between 2000 and 2016 in Ethiopia and their contribution to the reduction in diarrhoea and stunting in children under 5 years of age. Although progress has been made, improvements are still needed to increase the WASH standards and to address prowealthy and prourban inequalities in WASH coverage. Our findings showed that improvements in water and sanitation only modestly explained reductions in diarrhoea and stunting. More research is needed to identify other unexplored drivers of diarrhoea and stunting in Ethiopia.

## CONFLICTS OF INTEREST

The authors declare that they have no conflicts of interest. The opinions and statements in this article are those of the authors and may not reflect official policies or opinions of the organizations they belong to.

## CONTRIBUTIONS

MG and AH performed the research with help from TN, AS, MT and MH. AH analysed the data with help from TN and MG. MG and TN wrote the first draft of the paper. All authors took responsibility for final editing, reviewing and approval of the manuscript.

## Supporting information


**Table S1.** WHO/UNICEF JMP WASH Standards
**Table S2.** Description of Variables Used in the Analysis
**Table S3.** Change in outcome exposures and covariates between 2000 and 2016 among children aged 0–59 months
**Table S4.** Water, sanitation and child stunting and diarrhea in pooled (2000–2016) regression models^1^
**Figure S1.** Flow Diagram Showing Criteria Used to Include Children in Regression Analysis
**Figure S6.** Estimated contribution of the decline in surface water use and the practice of open defecation between 2000‐2016 to the decline in stunting observed over the same period

## Data Availability

All our analyses are based on Demographic and Health Surveys, which are available at the Measure DHS website after appropriate registration: http://dhsprogram.com/data/available-datasets.cfm.
