# Supplementary material for: Progress in Water, Sanitation and Hygiene (WASH) coverage and potential contribution to the decline in diarrhea and stunting in Ethiopia
Source: Matern Child Nutr. 2021 Nov 4;20(Suppl 5):e13280. doi: 10.1111/mcn.13280 (PMC11258769; doi:10.1111/mcn.13280)
Supplement: Supplementary file 1 — Table S1. WHO/UNICEF JMP WASH Standards Table S2. Description of Variables Used in the Analysis Table S3. Change in outcome exposures and covariates between 2000 and 2016 among children aged 0–59 months Table S4. Water, sanitation and child stunting and diarrhea in pooled (2000–2016) regression models1 Figure S1. Flow Diagram Showing Criteria Used to Include Children in Regression Analysis Figure S6. Estimated contribution of the decline in surface water use and the practice of open defecation between 2000‐2016 to the decline in stunting observed over the same period [file MCN-20-e13280-s001.docx]

**Table S1. WHO/UNICEF JMP WASH Standards**

| **Drinking Water Standards** | |
| --- | --- |
| Safely Managed | Drinking water from an improved water source that is located on premise, available when needed, and free from fecal and priority chemical contamination. |
| Basic | Drinking water from an improved source, provided collection time is not more than 30 minutes for a round trip, including queuing. |
| Limited | Drinking water from an improved source which collection time exceeds 30 minutes for a round trip, including queuing. |
| Unimproved | Drinking water from an unprotected dug well or unprotected spring |
| Surface Water | Drinking water directly from a river, dam lake, pond, stream, canal or irrigation canal. |
| Improved water sources include: piped water, boreholes or tube wells, protected dug wells, protected spring, rainwater, and packaged or delivered. | |
| **Sanitation Standards** | |
| Safely Managed | Use of improved facilities that are not shared with other households and where excreta are safely disposed of *in situ* or transported and treated off site. |
| Basic | Use of improved facilities that are not shared with other households. |
| Limited | Use of improved facilities shared between two or more households. |
| Unimproved | Use of pit latrines without slab or platform, hanging latrines or bucket latrines. |
| Open Defecation | Disposal of human feces in fields, forests, bushes, open bodies of water, other open spaces or with solid waste. |
| Improved facilities include: flush/pour flush piped to sewer system, septic tanks or pit latrines, ventilated improved pit latrines, composting toilets or pit latrines with slab. | |
| **Hygiene Standards** | |
| Basic | Availability of a handwashing facility on premise with soap and water. |
| Limited | Availability of a handwashing facility on premise without soap and water. |
| No Facility | No handwashing facility on premise. |
| Handwashing facilities may be fixed or mobile and include a sink with tap water, buckets with taps, tippy-taps, and jugs of basins designated for handwashing. Soap includes bar soap, liquid soap, powder detergent and soapy water but does not include ash, soil, sand, or other handwashing agents. | |

**Table S2. Description of Variables Used in the Analysis**

| **Variables** | **Definition** |
| --- | --- |
| Diarrhea | Percentage of living children (0-59 months) with diarrhea (three or more loose stools per day) at any time in the two weeks preceding the survey. |
| Stunting | Percentage of children (0-59 months) with height/length-for-age z score below -2 SD of the median based on the WHO 2006 Child Growth Standards. |
| Type of drinking water | Percentage of households whose main source of drinking water is basic, limited, unimproved or surface water. |
| Type of sanitation | Percentage of households who use toilet facilities that are basic, limited, unimproved or open defecation. |
| Household Wealth | EDHS calculated Wealth quintiles: Lowest, Second, Middle, Fourth, and Highest. |
| Maternal education | Percentage of women who attended any level of formal education. |
| Fathers education | Percentage of men who attended any level of formal education. |
| Maternal employment | Percentage of women who worked in the 12 months preceding the survey or are currently working. |
| Child age | Child age in months. (Dummy coded to include in the model) |
| Child Sex | Sex of child |
| Residence | Urban vs rural |
| Region | Region of residence |
| Survey round | Year of EDHS survey |

**Figure S1. Flow Diagram Showing Criteria Used to Include Children in Regression Analysis**

From the total number of children included in the four rounds of EDHS surveys (2000,2005,2011, and 2016), eligible children were those who are alive, are youngest children and those for whom anthropometric measurements were taken. Children with missing values for exposures and covariates were also removed from the analysis.

**Table S3. Change in outcome exposures and covariates between 2000 and 2016 among children aged 0-59 months**

| Variables | 0-5 months | | | | 6-11 months | | | 12-23 months | | | 24-59 months | | |
| --- | --- | --- | --- | --- | --- | --- | --- | --- | --- | --- | --- | --- | --- |
|  | 2000 | 2016 | P | 2000 | | 2016 | P | 2000 | 2016 | P | 2000 | 2016 | P |
|  | (n=907) | (n=973) |  | (n=920) | | (n=957) |  | (n=1746) | (n=1731) |  | (n=2798) | (n=2662) |  |
| Children |  |  |  |  | |  |  |  |  |  |  |  |  |
| Child sex: Female (%) | 48 | 51 | 0.386 | 49 | | 52 | 0.40 | 49 | 53 | 0.049 | 49 | 44 | 0.006 |
| Prevalence of diarrhea (%) | 15 | 6 | <0.001 | 39 | | 23 | <0.001 | 38 | 19 | <0.001 | 23 | 12 | <0.001 |
| Prevalence of Stunting (%) | 21 | 13 | <0.001 | 35 | | 17 | <0.001 | 61 | 39 | <0.001 | 70 | 49 | <0.001 |
| Maternal and paternal characteristics | |  |  |  | |  |  |  |  |  |  |  |  |
| Maternal height (cm) (mean(SD) | 156 (6.7) | 158 (6.6) | 0.001 | 157 (6.5) | | 157 (7.1) | 0.896 | 157 (6.6) | 157 (7.1) | 0.08 | 156 (7.6) | 157 (6.8) | 0.013 |
| Maternal formal education^1^ (%) | 19 | 40 | <0.001 | 17 | | 44 | <0.001 | 21 | 38 | <0.001 | 17 | 33 | <0.001 |
| Paternal formal education^1^ (%) | 41 | 52 | 0.001 | 36 | | 62 | <0.001 | 37 | 53 | <0.001 | 31 | 50 | <0.001 |
| Mother employed^2^ (%) | 60 | 36 | <0.001 | 59 | | 39 | <0.001 | 63 | 45 | <0.001 | 71 | 53 | <0.001 |
| Household characteristics |  |  |  |  | |  |  |  |  |  |  |  |  |
| Surface water use (%) | 41 | 13 | <0.001 | 40 | | 10 | <0.001 | 35 | 12 | <0.001 | 36 | 13 | <0.001 |
| Open defecation (%) | 85 | 38 | <0.001 | 86 | | 34 | <0.001 | 86 | 37 | <0.001 | 85 | 34 | <0.001 |
| Wealth |  |  |  |  | |  |  |  |  |  |  |  |  |
| Poorest (%) | 19 | 24 | 0.047 | 21 | | 20 | 0.584 | 22 | 24 | 0.076 | 23 | 20 | 0.017 |
| Poorer (%) | 20 | 23 | 0.123 | 24 | | 25 | 0.936 | 21 | 20 | 0.661 | 21 | 23 | 0.406 |
| Middle (%) | 25 | 19 | 0.024 | 19 | | 22 | 0.14 | 22 | 23 | 0.694 | 19 | 20 | 0.396 |
| Richer (%) | 22 | 19 | 0.194 | 24 | | 17 | 0.023 | 20 | 19 | 0.541 | 19 | 19 | 0.541 |
| Richest (%) | 15 | 15 | 0.969 | 13 | | 17 | 0.108 | 17 | 14 | 0.164 | 18 | 18 | 0.936 |

^1^ Attended any level of formal education. ^2^ Women who worked in the 12 months preceding the survey or are currently working.

**Table S4. Water, sanitation and child stunting and diarrhea in pooled (2000 -2016) regression models^1^**

|  | N | Surface Water  Beta [95% CI] | *p* | Open Defecation  Beta [95% CI] | *p* |
| --- | --- | --- | --- | --- | --- |
| Diarrhea |  |  |  |  |  |
| 0-5 months | 2996 | 0.04 [0.01,0.07] | 0.018 | 0.03 [-0.00,0.06] | 0.087 |
| 6-11 months | 3054 | -0.01 [-0.05,0.03] | 0.707 | -0.01 [-0.05,0.03] | 0.700 |
| 12-23 months | 5562 | 0 [-0.03,0.03] | 0.857 | 0 [-0.04,0.03] | 0.869 |
| 24-59 months | 8897 | -0.01 [-0.03,0.01] | 0.220 | 0.03 [0.01,0.05] | 0.012 |
| 0-59 months | 20509 | 0 [-0.02,0.01] | 0.816 | 0.02 [-0.00,0.03] | 0.063 |
| Stunting |  |  |  |  |  |
| 0-5 months | 2988 | 0.02 [-0.01,0.05] | 0.206 | 0.01 [-0.02,0.04] | 0.489 |
| 6-11 months | 3045 | -0.01 [-0.04,0.03] | 0.631 | 0.04 [-0.00,0.07] | 0.057 |
| 12-23 months | 5534 | 0.01 [-0.03,0.04] | 0.709 | 0.03 [-0.00,0.07] | 0.056 |
| 24-59 months | 8841 | 0.01 [-0.02,0.03] | 0.663 | 0.03 [-0.00,0.06] | 0.064 |
| 0-59 months | 20408 | 0 [-0.01,0.02] | 0.729 | 0.03 [0.01,0.05] | < 0.001 |

^1^ Beta coefficients (95% CI) are estimated using linear probability regression model with a robust variance estimator. Models were adjusted for maternal education, paternal education, maternal employment, wealth, region, residence, age, sex and survey round.

**Figure 6. Estimated contribution of the decline in surface water use and the practice of open defecation between 2000-2016 to the decline in stunting observed over the same period**

**Figure 6. Estimated contribution of the decline in surface water use and the practice of open defecation between 2000-2016 to the decline in stunting observed over the same period**

Percent contribution of the change in surface water use and open defecation to the decline in stunting, 2000-2016. The percent contribution of factors was estimated using simple decomposition in which changes in the percentages of the exposures and covariates are multiplied by the pooled regression coefficients. Other category includes maternal and paternal education, maternal height, household wealth, maternal employment, child age, child sex, residence, region and survey round.
